# Reply: “Comment on ”Consumption of Cooked Black Beans Stimulates a Cluster of Some Clostridia Class Bacteria Decreasing Inflammatory Response and Improving Insulin Sensitivity.” *Nutrients* 2020, *12*(4), 1182”

**DOI:** 10.3390/nu12072093

**Published:** 2020-07-15

**Authors:** Mónica Sánchez-Tapia, Irma Hernández-Velázquez, Edgar Pichardo-Ontiveros, Omar Granados-Portillo, Amanda Gálvez, Armando R. Tovar, Nimbe Torres

**Affiliations:** 1 Departamento de Fisiología de la Nutrición, Instituto Nacional de Ciencias Médicas y Nutrición Salvador Zubirán, Mexico City 14080, Mexico; qfbmoniktc@gmail.com (M.S.-T.); edgar.pichardo@gmail.com (E.P.-O.); ograpo@yahoo.com (O.G.-P.); armando.tovarp@incmnsz.mx (A.R.T.); 2 Facultad de Química, Universidad Nacional Autónoma de México, Mexico City 0410, Mexico; hernandezvelazquezia@gmail.com (I.H.-V.); galvez@unam.mx (A.G.)

We appreciate the interest of Dr. Cervantes for his/her comments [1] on our article [2] and for providing us with the opportunity to extend the discussion regarding diet composition in our interpretation of the findings of gut microbiota and fecal butyrate concentration. The following is our response to his/her concerns.

The composition of the **control diet** in our study was based on recommendations made by the American Institute of Nutrition in 1993 [3], which was designed to provide all the nutrients to meet the requirements of the rodent. **Protein:** The control diets contained 17% dietary protein using casein as the reference protein, and in the black bean group, the amount of black bean protein present in the cooked dry black bean was adjusted to achieve the same concentration as the control group. **Fat:** The recommended amount of fat in the control diet is 7% soybean oil as the reference fat. For the black bean group, the fat in the cooked dry black bean was measured and the total fat amount was adjusted to 7% with soybean oil as recommended. **Total carbohydrates:** The recommended carbohydrates in the diet are cornstarch, dextrinized cornstarch, and sucrose. Total carbohydrates and fiber constituted 69.3 g/100 g of the casein diet, while they made up 70.9 g/100 g of the black bean diet. Since 44% of the carbohydrates in the cooked black bean are primarily starch; the percentage of carbohydrates in the diet was adjusted with proportional amounts of dextrinized cornstarch and sucrose to adjust for the percentage of carbohydrates. For this reason, there was a difference in sucrose content in the control diets. Therefore, the diets had similar energy content (3.95 kcal/g).

For **high-fat diets**, casein or black bean diets with 5% sucrose in drinking water contained 24% fat and 24% protein. High-fat diets with both types of protein provided approximately 4.8 kcal/g. It is important to note that the difference in sucrose consumption was less evident in the high-fat groups, since both the casein and black bean groups had sucrose in the drinking water. In fact, the cooked dry black bean group consumed more sucrose in the water (20–25 mL/day) than the casein group (12–15 mL/day). However, despite the difference in sucrose consumption in drinking water, the metabolic effects of cooked dry black beans were still present, suggesting that not only sucrose is the differential factor in the biological effects observed but also that other factors such as the presence of resistant starch and the type of protein are responsible of the changes in gut microbiota that can in part determine the changes in body weight and body fat percentage, leptin levels, and blood glucose response. In fact, the improved glucose response in groups fed cooked dry black beans may be associated with specific changes in intestinal microbiota, in particular an increase in the genus *Prevotella* [4].

Interestingly, we observed the same proportion of Firmicutes/Bacteroidetes in all groups; however, there were differences in circulating lipopolysaccharide (LPS) concentrations between groups fed casein diets and those fed black bean diets. Analysis of LDA score indicated that the bacteria that increased significantly in the group fed casein high-fat diet was *Bacteroides eggerthii*, a Gram (-) bacteria, and this group showed the highest concentration of LPS in serum, while the group fed black bean high-fat diet showed a significant increase in *Butyricicoccus pullicaecorum* and *Ruminicoccus callidus,* which are Gram (+) bacteria, and showed the lowest serum concentration of LPS. Thus, the ratio Firmicutes/Bacteroidetes is not always associated with metabolic endotoxemia as has been recently suggested [5].

In addition, a decrease in the abundance of proteins involved in the formation of tight junctions, including occludin, is associated with an increase in intestinal permeability and metabolic endotoxemia. Although we did not perform a permeability test, we demonstrated that animals fed a casein high-fat diet showed a lower abundance of the protein occludin than the group fed a black bean high-fat diet. We found a significant inverse correlation between Log LPS and the abundance of occludin (*p* = 0.0001; *r* = 0.9267).

Fermentation of substrates by the intestinal microbiota produces, among other products, short-chain fatty acids (SCFA). In our study, we found that total fecal SCFA in groups fed casein diet or casein high-fat diet were higher than those fed black bean diets. However, the black bean groups showed the highest concentration of butyrate, compared to the groups fed casein diets. Butyrate is known to promote epithelial barrier function by inducing genes encoding tight junctions [6]. Therefore, consumption of the black bean diet had a selective effect on the formation of SCFA.
